# Titanium dioxide nanostructure-loaded Adriamycin surmounts resistance in breast cancer therapy: ABCA/P53/C-myc crosstalk

**DOI:** 10.2144/fsoa-2023-0107

**Published:** 2024-05-15

**Authors:** Rehab M Abdel-Megeed, Abdel-Hamid Z Abdel-Hamid, Mai O Kadry

**Affiliations:** 1 Therapeutic Chemistry Department, Pharmaceutical & Drug Industries Research Institute, National Research Center, El Buhouth St., Dokki, Cairo, 12622, Egypt

**Keywords:** ABCA1, Adriamycin, breast cancer, C-myc, drug resistance, titanium dioxide nanoparticles

## Abstract

**Aim:** To clarify the alternation of gene expression responsible for resistance of Adriamycin (ADR) in rats, in addition to investigation of a novel promising drug-delivery system using titanium dioxide nanoparticles loaded with ADR (TiO2-ADR). **Method:** Breast cancer was induced in female Sprague-Dawley rats, followed by treatment with ADR (5 mg/kg) or TiO_2_-ADR (2 mg/kg) for 1 month. **Results:** Significant improvements in both zinc and calcium levels were observed with TiO_2_-ADR treatment. Gene expression of ATP-binding cassette transporter membrane proteins *(ABCA1 & ABCG1)*, *P53* and *Jak-2* showed a significant reduction and overexpression of the C-myc in breast cancer-induced rats. TiO_2_-ADR demonstrated a notable ability to upregulate these genes. **Conclusion:** TiO2-ADR could be a promising drug-delivery system for breast cancer therapy.

## Plain language summary

The current study aimed to investigate a novel and promising drug-delivery system to overcome the resistance problem by loading Adriamycin (ADR) into titanium dioxide nanoparticles (TiO_2_). The study also aimed to clarify the changes in gene expression responsible for the development of ADR resistance, in a rat model. First, animals were divided into four groups of ten each. Breast cancer was induced in female Sprague-Dawley rats by administering two doses of DMBA (50 and 25 mg/kg), followed by treatment with ADR at a dose of 5 mg/kg for 1 month, or TiO_2_-ADR at a dose of 2 mg/kg for 1 month. Biochemical and molecular analyses were conducted. Zinc and calcium levels were found to significantly decrease after cancer induction. Treatment with ADR alone or in combination with TiO_2_ showed a significant improvement in both mineral levels, with the TiO_2_-ADR group showing superior results. Gene expression of ATP-binding cassette transporter membrane proteins (*ABCA1* & *ABCG1*), *P53* and *Jak-2* showed a significant decrease after DMBA-induced breast cancer. However, both the ADR- and TiO_2_-ADR-treated groups showed a notable increase in gene expression, with the TiO_2_-ADR group showing the highest increase. On the other hand, there was a significant overexpression of the *C-myc* gene after DMBA-induced breast cancer. However, both ADR and TiO_2_-ADR treatments resulted in a notable decrease in *C-myc* gene expression. Based on the data, TiO_2_-ADR could be a promising drug-delivery system for breast cancer therapy.

## Abstract (plain language summary)

Breast cancer induced by DMBA in rat model.

Intoxicated rats treated by standard drug Adriamycin alone or loaded into TiO_2_.

Genes related to resistance were estimated in all studied groups.

Data declared the superiority of TiO_2_-ADR in modulation gene expression as well as biochemical parameters.

## Graphical abstract

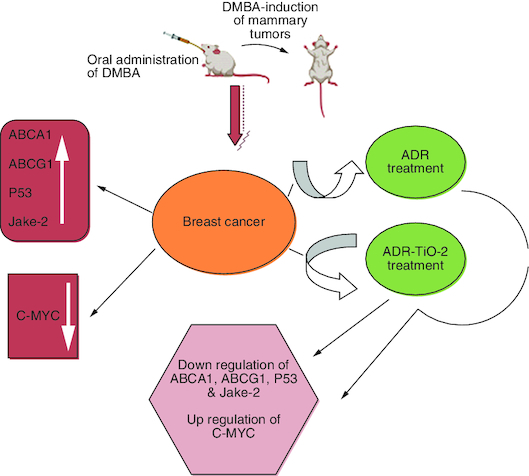

Breast cancer has the highest incidence and mortality rate among female malignancies, surpassing lung cancer worldwide. According to the world cancer statistics of 2023, one in every eight women (nearly 13%) is diagnosed with breast cancer [1]. The therapeutic strategies for breast cancer treatment are determined by the biological properties of the tumor. Hormone treatment may be indicated if the tumor is low grade, node-negative and estrogen-receptor positive. However, chemotherapy is often given before targeted therapies if the tumor is high grade and/or node positive [2,3]. Understanding how cells respond to chemotherapy drugs can lead to the discovery of successful cancer therapies [4]. One of the major factors contributing to treatment failure is the ability of cancer cells to acquire therapeutic resistance against cytostatic medicines. Understanding the processes underlying cancer cell behavior, including drug resistance, would presumably have a significant impact on the effectiveness of future therapeutic interventions [5]. Adriamycin (ADR), one of the various medications used in anticancer therapy, is the one that frequently receives a lot of attention due to its widespread usage in different tumors treatment. ADR belongs to anthracyclines, which are currently used for combination adjuvant breast cancer treatment [6]. ADR may lead to drug resistance and in hence tumor development, which is negatively affecting the prognosis and survival of patients [7]. Despite numerous studies on various pathways, ADR's resistance remains an unresolved issue in cancer therapy. The interplay between signaling pathways has been investigated as a potential mechanism for inducing ADR resistance through the promotion of proliferation, cell cycle development and inhibition of apoptosis [8]. ADR's mechanisms of anti-mitotic and cytotoxic interaction have been commonly associated with the induction of DNA damage, primarily through the inhibition of topoisomerase II, intercalation between base pairs, direct membrane effects and the production of reactive oxygen species (ROS), which leads to DNA damage and lipid peroxidation [9]. Furthermore, ADR inhibits the activity of polymerase and influences regulation of gene expression [10].

The underlying causes of chemo-resistance are multifaceted and include both drug- and tumor-related factors [11,12]. One potential mechanism involves the overexpression of ATP-binding cassette (ABC) transporter membrane proteins, which can reduce levels of chemotherapeutic drugs in cells [13]. Despite the fact that research involving breast cancer has revealed that ABC transporters are related with decreased survival and treatment resistance, the results have been very inconsistent [14]. A super family of integral membrane proteins known as ABC transporters is widely distributed and is responsible for the ATP-powered translocation of substrates across membranes [14]. ABC transporters are classified into seven subfamilies (*ABCA–ABCG*), with 49 ABC transporters [13]. Drug resistance in cancer has been linked to four subfamilies (*ABCA, ABCB*, *ABCC* and *ABCG*) [13]. This study investigated *ABCA1* and *ABCG1* in connection to breast cancer chemoresistance [15].

The tumor suppressor gene *p53* is an essential transcription factor [16]. Functionally, activating *p53* coordinates an intricate anti-proliferative transcriptional pathway, which in turn causes a diverse range of biological reactions [17]. When *p53* is activated, many genes involved in the cell division cycle are suppressed. For example, cyclin family members and their kinase are inhibited upon elevation of *p53* levels [18]. Moreover, apoptosis is an important consequence of *p53* overexpression [19]. *p53* activation initiates *BAX, PUMA* and *NOXA*, then *p53* is attached to the pro-apoptotic *Bcl-2*, which is situated on mitochondria and triggering endogenous apoptosis through cytochrome c/ *Apaf-1* [20]. The *p53* tumor suppressor gene is mutated in various cancer types, including breast cancer [21,22].

*C-myc*, a proto-oncogene, plays an essential role in cellular metabolism [23], apoptosis [24], differentiation [25], cell cycle progression [26] and cancer [27]. The gene *C-myc* encodes a basic helix-loop-helix leucine zipper transcription factor that transcribes a variety of downstream target genes. *C-myc* is aberrantly expressed in multiple human solid malignancies and is an attractive target for cancer chemotherapeutics [28]. Previous studies have associated *C-myc* overexpression with chemotherapeutic resistance in salivary carcinoma. Inhibition of *C-myc* has been shown to reverse drug resistance in several malignancies, including lung cancer and melanoma [28]. *C-myc* antisense oligodeoxy nucleotides have been found to enhance the sensitivity of cisplatin in metastatic melanoma cell lines [28]. However, while *C-myc* has been shown to be beneficial in the treatment of cancers such as hepatocellular carcinoma and leukemia, its main role in drug resistance has not yet been elucidated [29].

Micronutrients such as minerals also play an essential role in cellular metabolism and are linked to numerous pathological conditions as well as oncogenesis [30]. Although previous research on the relationship between zinc in blood and diet, and the risk of breast cancer has been inconclusive, zinc has been implicated in the development of the disease [30]. Moreover, zinc is a crucial trace element as it serves as a cofactor for more than 300 enzymes, supporting the growth and maintenance of our body. Numerous physiological processes, including synthesis of DNA and RNA, cell proliferation, division and apoptosis, are controlled by it. While its role in malignancy progression is pivotal, the mechanism is not yet fully understood [31]. The relationship between blood zinc values and risk of breast cancer was previously investigated [32]. Additionally, distinct patterns of zinc concentration and the zinc transportation network have been observed in breast cancer.

Calcium, an important mineral, directly participates in cell-to-cell adhesion and is a second messenger in several signaling pathways as cell cycle and cellular proliferation [33]. Furthermore, it plays a role in the folding of numerous enzymes synthesized in the endoplasmic reticulum such as calnexin which are linked to different diseases including cancer [34]. Different previous studies suggested the effect of cancer on mineral concentration. Furthermore, several studies linked between oxidative stress and calcium levels in cardiac damage in all references were mentioned.

Titanium dioxide nanoparticles (TiO_2_) are well-known nanoparticles that have been extensively studied in both *in vitro* and *in vivo* settings. They possess unique surface chemistry and morphologies, such as different sizes and shapes. TiO_2_ nanoparticles exhibit good biocompatibility and possess inherent biological activities, including effective antimicrobial and antitumor properties, with minimal side effects [35]. According to earlier research, TiO_2_ disrupts the epidermal growth factor receptor signaling cascade by inducing ROS-mediated cytotoxicity and genotoxicity. These are the main underlying molecular processes that cause cell death in malignant cells, while nearby normal cells are less affected [36]. However, the relative therapeutic impact of TiO2 for breast cancer in comparison to more traditional treatments, such ADR, remains unknown. ADR is currently one of the most effective anticancer medications available [35]. The clinical application of ADR is limited by harmful side effects, particularly cardiotoxicity, which can lead to cardiomyopathy and congestive heart failure. Decreased levels of antioxidants contribute to increased oxidative stress and ROS production, damaging the heart muscle [37]. Additionally, altered levels of Ca^2+^ ions play a role in ADR-induced cardiotoxicity and result in apoptosis.

The current study discusses drug-resistance problem associated with ADR. We selected ADR (common name is doxorubicin) as it is the common standard drug in Egyptian marketing for breast cancer treatment. Based on these observations, efforts have been made to develop novel drug-delivery systems, such as TiO_2_, which is chemically stable, nontoxic and environmentally friendly. The goal of the current study is to enhance the chemotherapeutic efficiency of ADR-TiO_2_ against a breast cancer tin rat model. Furthermore, this study extended to investigate signaling pathways crosstalk in breast cancer and impact of the standard drug ADR as well as TiO_2_-ADR in gene expression modulation of the interrupted genes as a promising drug-delivery system to overcome resistant problem associated with ADR treatment in breast cancer therapy.

## Materials & methods

### Chemicals

7,12-dimethylbenz(a) anthracene (DMBA), ADR and TiO_2_ were purchased from Sigma-Aldrich Co. (MO, USA). Specific primers for the expression of *C-myc, ABCA1, ABCG1* and *P53* genes were purchased from Thermo Fisher Scientific (MA, USA). Both total RNA extraction kits and SYBR green RT-PCR kits were purchased from Qiagen (Helden, Germany). Kits for determination of both zinc as well as calcium minerals were obtained from Biodiagnostic (Giza, Egypt). All reagents and chemicals were of high analytical integrity.

### Synthesis of Tetanium dioxide nanoparticle-loaded Adraimycin 

Approximately 0.3 ml of ADR (1 mg/ml^-1^) was added into 10 ml of Boron-doped TiO_2_ (B-TiO_2_) (0.75 mg/ml^-1^) and nanocomposite (NC) (0.75 mg/ml^-1^) then stirred for 24 h. ADR loaded nano-carriers (ADR-TiO_2_) were collected after centrifugation and stored in Phosphate buffer saline (pH 7.4) [37].

### Animals & breast cancer induction

40 female Sprague-Dawley rats (110 g ± 10), were obtained from National Research Center in Egypt. They were maintained in an optimum conditions (23 ± 5 °C; 50 ± 5% humidity; 12 h/12 h dark/light cycle). They were also freely provided with ordinary diet and water. In this study, animals received proper care and handling in compliance with the institutional animal ethics committee of the National Research Centre, Egypt (approval no. 19075).

The ability of animals to get their food and water was observed daily in the morning for measuring pain and suffering in addition to outward appearance. Furthermore, changes of disease and behavior were also concerned. Rats were also observed for urine/fecal output before beginning cleaning and any signs of vomiting.

Rats were randomly divided into four groups of ten animals each. Group (1): animals received normal saline and served as control. Groups (2, 3 and 4) received (50 mg/kg) DMBA in olive oil by oral gavages [38]. All rats were palpated twice weekly to detect mammary tumors. After 6 weeks, the rats were given a booster dose of BMBA (25 mg/kg). Group (2): untreated animals were served as positive control group. Group (3): animals were treated with intraperitoneal injection by ADR at a dose of 5 mg/kg for 1 month [10]. Group (4): treated animals with intraperitoneal injection by ADR-TiO_2_ at a dose of 2 mg/kg for 1 month [37]. Two out of ten animals died in the ADR-treated group, four animals died in the positive control untreated animals group.

### Blood & tissue sampling

In order to count tumors, examine their morphology and determine the largest diameter of tumors, hairs were extracted by shaving the area around the tumor and then the tumor diameter was measured. The largest tumor was used to calculate the maximum diameter, which was measured directly for single tumors (tumor size range recorded 9 mm ± 2 in size).

At the end of the current experiment, rats were subjected to anesthesia using carbon dioxide method and followed by cervical dislocation for euthanasia. After that, blood samples were collected from the sublingual vein and centrifuged at 4000 rpm for 15 min. The obtained sera were kept at -20°C for biochemical determinations. Rats were sacrificed by cervical dislocation and the breast samples were removed and washed with saline. The tissue was frozen at -80 °C for RNA extraction and gene expression study.

### Estimation of calcium & zinc

Calcium and zinc minerals were estimated in serum samples using Biodiagnostic kits according to the manufacturer's instructions. In an alkaline media, calcium ions were reacted with methylthymol blue to generate a blue color and its intensity was proportional to the calcium concentration. The interference caused by the magnesium ions was eliminated when hydroxy 8-quinoline was present. Calcium concentration in serum sample was measured at a wavelength 585 nm. Furthermore, at alkaline pH, zincon (2-caboxy-2′-hydroxy-5-Sulfoformazyl-benzene) in the reagent chelates zinc in the sample. This complex's production is detected at a wavelength of 610 nm [39,40].

### Quantitative RT-PCR analysis

Total RNA extraction was carried out using QIAamp mini kit (Qiagen; CA, USA; cat number 74104) form breast tissue according to the manufacturer's instructions. Gene expression of *ABCA1, ABCG1, Jak-2, P53* and *C-myc* was performed using one step QuantiTecht SYBR green RT-PCR Master Mix (Qiagen; cat number 204243). The reaction was run using StratageneMx3000 P QPCR System (Agilent Technologies, CA, USA). The sequences of primers are described in Table 1. The temperature profile was as follows: 94 °C for 3 min, 94 °C for 20 s, 47–57 °C (according to the optimum annealing temperature of each primer) for 20 s and 72 °C for 10 s. PCR cycles were repeated 40 times. The relative expression of target genes was obtained using comparative CT (2^-ΔΔCT^) method against β-actin as a reference gene [41,42].

**Table 1.** Sequence of forward and reverse primers.

Primer name	Forward	Reverse
*ABCA1*	5′-GGGTGGCTTCGCCTACTTG-3′	5′-GACGCCCGTTTTCTTCTCAG-3′
*ABCG1*	5′-GTACTGACACACCTGCGAATCAC-3′	5′-TCGTTCCCAATCCCAAGGTA-3′
*P53*	5′-CTGTCATCTTCTGTCCCTTC-3′	5′-TGGAATCAACCCACAGCTGCA-3′
*Jak-2*	5′-GCAGTGACCTCCAGAGACAGTCTATCTTTGAAGCAATACGTATGA-3′	5′-GCAGTGACCTCCAGAGACACTTACTTCGTCTCCACAGAA-3′
*C-myc*	5′-ATCACAGCCCTCACTCAC-3′	5′-ACAGATTCCACAAGGTGC-3′
*β-actin*	5′-CTTTGATGTCACGCACGATTTC-3′	5′-GGGCCGCTCTAGGCACCAA-3′

### Statistical analysis

Data were expressed as means ± SD. Statistical analysis was carried out using one-way analysis of variance (ANOVA) and Tukey's post-HOC test. The level of significance was set at p < 0.05.

## Results

### Modulation of calcium & zinc concentration in serum

DMBA-induced breast cancer in rats declared a significant reduction in calcium concentration as well as zinc recording 10.59839 mg/dl and 203.69 μg/dl, respectively as compared with negative control. Data recoded declared non-significant improvement in calcium concentration upon ADR treatment. On the other hand, a significant improvement in the concentration of calcium elucidated upon treatment by TiO_2_-ADR recoding 10.16887 mg/dl.

However treatment by ADR as well as TiO_2_-ADR elucidated a significant improvement in zinc concentration with the superiority of TiO_2_-ADR in the improving of the interrupted minerals' concentration (Tables 2 & 3) Data were expressed as means ± SD (n = 10). p < 0.05 is considered significant.

**Table 2.** Calcium concentration in breast cancer serum samples post treatment by Adriamycin and combination of both TiO_2_ and Adriamycin.

Groups	Calcium concentration (mg/dl) ± SD
Negative control	10.61 ± 0.56^(a)^
Positive control	8.09 ± 0.75^(b)^
Doxorubicin	8.44 ± 0.63^(b)^
TiO_2_-Dox	10.17 ± 0.35^(a)^

Data are expressed as means ± SD (n = 10). p < 0.05 is considered significant. Groups having the same letter are not significantly different from each other, while those having different letters (a, b, c & d) are significantly different from each other.

**Table 3.** Zinc concentration in breast cancer serum samples post treatment by Adriamycin and combination of both TiO_2_ and Adriamycin.

Groups	Zinc concentration (μg/dl) ± SD
Negative control	203.69 ± 5.78^(a)^
Positive control	96.92 ± 6.14^(b)^
Doxorubicin	130.55 ± 4.28^(c)^
TiO_2_-Dox	192.64 ± 6.14^(a)^

Data are expressed as means ± SD (n = 10). p < 0.05 is considered significant. Groups having the same letter are not significantly different from each other, while those having different letters (a, b, c & d) are significantly different from each other.

### Modulation of *ABCA1* & *ABCG1* gene expression

**Figure 1.**
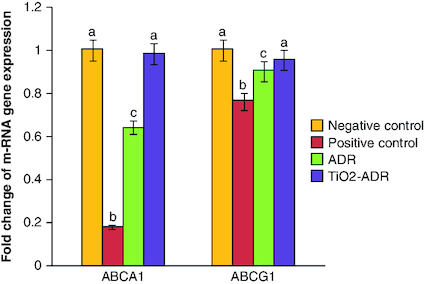
Effect of Adriamycin and combination of both TiO_2_ and Adriamycin against breast cancer in rat breast tissue on *ABCA1* and *ABCAG1* gene expression. Data were expressed as means ± SD (n = 10). p < 0.05 is considered significant. Groups having the same letter are not significantly different from each other, while those having different letters are significantly different from each other.

Breast cancer induction declared a significant reduction in the expression of *ABCA1* gene (0.18-fold change) as compared with negative control group (onefold change). On the other hand, a significant elevation in the expression of *ABCA1* was noticed upon ADR and TiO_2_–ADR treatments recording 1.24-, 0.64- and 0.98-fold changes, respectively (Figure 1). Results declared the superiority of TiO_2_–ADR in modulating genes responsible for resistance. Meanwhile a significant change in the expression of *ABCG1* gene was declared upon DMBA-induced breast cancer in rats recording 0.76-fold change as compared with healthy rats. Furthermore, a nonsignificant modulation was declared in all treated regimens to be near the normal value recording 0. 91, 0.91 and 0.95-fold change (Figure 1). Data were expressed as means ± SD (n = 10); p < 0.05 is considered significant.

### Regulation of *Jak-2* & *P53* gene expression

**Figure 2.**
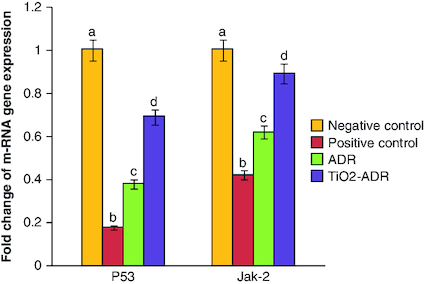
Effect of Adriamycin and combination of both TiO_2_ and Adriamycin against breast cancer in rat breast tissue on *P53* and *Jak-2* gene expression. Data were expressed as means ± SD (n = 10). The p < 0.05 is considered significant. Groups having the same letter are not significantly different from each other, while those having different letters are significantly different from each other.

A significant reduction in *P53* as well as *Jak-2* gene expression was elucidated post DMBA intoxication recording 0.18- and 0.42-fold changes as compared with negative control group. Meanwhile, treatment with ADR and TiO_2_–ADR declared a significant increment in the expression of target genes noticed the superiority of TiO_2_–ADR to modulate the inhibition of *Jak-2* gene expression recording 0.92-fold changes (Figure 2) Data were expressed as means ± SD (n = 10); p < 0.05 is considered significant.

### Modulation of *C-myc* gene expression

**Figure 3.**
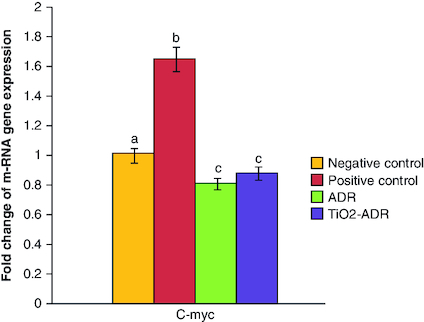
Effect of Adriamycin and combination of both TiO_2_ and Adriamycin against breast cancer in rat breast tissue on *C-myc* gene expression. Data were expressed as means ± SD (n = 10). p < 0.05 is considered significant. Groups having the same letter are not significantly different from each other, while those having different letters are significantly different from each other.

The induction of breast cancer elucidated a significant elevation in the expression of *C-myc*; the responsible gene for resistance in cancer therapy (1.65-fold change) as compared with negative control group. On the other hand, the expression of *C-myc* gene expression was significantly downregulated upon ADR and TiO_2_–ADR treatments recording 0.75-, 0.45- and 0.86-fold changes, respectively (Figure 3). Results declared the superiority of TiO_2_–ADR in modulating C-myc gene expression. Data were expressed as means ± SD (n = 10); p < 0.05 is considered significant.

## Discussion

ADR is still one of the most active and extensively used chemotherapeutic medicines for treating early and advanced breast cancer. Nevertheless, tumor resistance has reduced the agent's usefulness in single-drug therapy regimens. The precise processes behind resistance remain unknown, with *in vitro* research utilizing breast cancer cell models frequently lacking therapeutic relevance.

Despite numerous pathways being investigated, ADR resistance remains a major unsolved issue in cancer therapy [4]. Studies have shown that interaction between signaling pathways can enhance resistance to the drug by inducing cell proliferation, progressing the cell cycle and preventing apoptosis [43].

The current observation declared a significant alternation in calcium and zinc levels upon breast cancer induction. Different previous investigations reported that calcium and zinc are significantly higher in breast cancer cases [44–46]. Furthermore, calcium concentration in breast cancer tissue is associated with micro-calcifications which have significant radiological features for localization of tumor cells. Zinc has direct impacts on malignant cells, including changes in zinc transporter expression, the stability and performance of intracellular machinery related to cell division, immunological function and also involved in apoptosis [32,47].

In the current study, DMBA-induced breast cancer in murine model could alter signaling pathways responsible for resistance. Herein *ABCA1* and *ABCG1* gene expression was reduced upon DMBA intoxication. Meanwhile treatment by TiO_2_-ADR elucidated more significant impact than treatment by ADR on modulating *ABCA1* and *ABCG1* gene expression.

Drug transporters such as *ABCA1* play a critical role in pretargeted drug resistance, which elucidates the ability of cancer cell to adapt to different drugs by increasing drug production and decreasing drug absorption [48]. Preventing the production of ABC transporters in cancer cells may help prevent drug resistance, as studies in the past have indicated that chemotherapy failure is directly tied to these proteins [49]. Several anticancer medications are carried outside of cells and fail to exert their anticancer effects because cancer cells have a high expression of transporters. *ABCA1* previously was shown to be expressed in ADR-sensitive triple-negative breast cancer cells and in ADR-sensitive osteosarcoma cells [50]. Furthermore, Wang, *et al.*, investigated the association between the expression ABCA1 gene and the development of resistance against chemotherapy in ovarian cancer [51].

However, several investigations have revealed that *ABCA1* is a tumor suppressor gene that encourages cholesterol metabolism for the inhibition of cancer progression (26–28). *ABCA1* was previously revealed to have antitumor effects and expresses in low level in colon cancer. As the expression of insufficient quantity of *ABCA1* leads to accumulation of cholesterol in cancer cells, prevent releasing of mitochondrial *TNF*, and hence accelerates the development of the disease [52].

Furthermore, P53 as an important tumor-suppressor gene possess an essential role in the progression of cancer by causing cell cycle arrest, DNA repair or apoptosis, *p53* inhibits the growth of tumors and provides protection against DNA damage [53,54]. In our study, *P53* gene expression was reduced post DMBA intoxication. The *p53* pathway, on the other hand, is frequently mutated in cancer [54]. *TP53* gene mutations or deletions are seen in about 50% of human malignancies, largely resulting in decreased tumor suppressor activity [55]. Damaged cells may multiply after *p53* functioning is lost [55]. Drug resistance is caused by mutations in the *p53* gene that alter the pro-apoptotic balance. A significant study carried out by the National Cancer Institute, in which 60 cell lines and more than 100 anticancer drugs were examined, has confirmed the relationship between mutant *p53* and sensitivity to anticancer drugs [56].

*JAK-2*, a member of the JAK family of protein tyrosine kinases, is a crucial intracellular modulator of cytokine and hormone signaling. Different earlier investigations revealed that the *JAK-2* signaling pathway is linked to the development of cancer [57]. Upregulation of *JAK-2* in breast cancer enhances survival and growth of cancer cell by regulating of cell division and apoptosis through overexpression of *Bcl-2* family members and cyclin [58,59].

Clinically, amplification of *JAK-2* in breast cancer samples is highly linked to poor in overall survival, and low response to chemotherapy [60]. Due to the conclusive role of the *JAK-2* signaling pathway in breast cancer, various researches were conducted on breast cancer therapy through targeting *JAK-2* by gene inhibitors [59].

The data revealed in the current study recorded a significant elevation in *C-myc* gene expression upon DMBA induced breast cancer.

We recorded that overexpression of *C-myc* was a characteristic feature in drug-resistant cells. As previously investigated, overexpression of *C-myc* reduced the sensitivity of the drug. These records clearly proved an essential role of *C-myc* in the drug resistance and declared that *C-myc* inhibition could be a promising to overcome drug resistance in breast cancer therapy.

Numerous previous investigations reported that overexpression of *C-myc* enhances tumor progression *in vitro* and *in vivo* while its downregulation can arrest tumor cell growth, by enhancing apoptosis [61]. Clinical research indicated that cancer patients have a significant elevation in *C-myc* gene expression compared with healthy individuals [61,62].

On the other hand, TiO_2_-ADR treatment recorded a notable improvement in drug resistance as well as modulation in all interrupted genes upon cancer induction. ADR-TiO_2_ may target tumor cell due to the encapsulation of doxorubicin in titanium dioxide nanoparticles. Furthermore, this formula enables to reduce the doxorubicin dose and therefore ameliorates its toxic side effect, especially cardiotoxicity and overcome drug resistance problem.

Based on these findings, several studies were conducted to investigate a novel drug-delivery system based on the loading of ADR into TiO_2_ to enhance ADR chemotherapeutic efficiency, reduce its side effects and solve drug resistance problem [36]. It was reported that TiO_2_ could induce apoptosis in breast cancer cell by interfering signaling pathways TiO_2_ cause ROS generation which activates the release of pro-inflammatory cytokines release such as *TNF-α* [63]. TiO_2_ could induce p53 and activate cell-cycle checkpoint kinases [64]. Furthermore, TiO_2_ could induce DNA damage in HepG2 cells [65] Furthermore, previous investigations confirmed the potential effect of doxorubicin-loaded nanoparticles in tumor therapy [66,67]. These studies all indicate that targeting oxidative stress induced in cancer cell is the key mechanism of TiO_2_.

## Limitations of the study

The study concerned with resistance drug problem associated with the most common chemotherapeutic drug, doxorubicin and study signaling pathways responsible for resistance. Furthermore, the study investigated a novel drug-delivery system through loading ADR in TiO_2_ nanoparticles. In a later study, the impact and safety of nanostructure-loaded Adriamycin (TiO_2_-ADR) will be discussed as a promising candidate for breast cancer therapy.

## Conclusion

In summary, breast cancer induced animals demonstrated a significant alternation in both mineral concentrations and gene expression of signaling pathways responsible for resistance in chemotherapeutic strategy of ADR. Furthermore, improved recommendations for drug research initiatives may result from an understanding of how chemoresistance develops in breast cancer cells in more physiologically relevant models. However, the clinical application of ADR is limited due to its harmful side effects, it is urgent to investigate safer and more effective anticancer drug. Our study established the vital role of TiO_2_-ADR in breast cancer therapy. We also identified TiO_2_-ADR as a therapeutic target for breast cancer treatment and as a promising candidate to reduce drug resistance problem associated with ADR in addition to improve its therapeutic efficacy. In order to enhance characterization of the mechanisms involved in TiO_2_-ADR for breast cancer treatment, additional studies should be conducted.
